# A View of the Hospital Arrangements

**Published:** 1914-08-15

**Authors:** 


### The East Coast and the War.

### A VIEW OF THE HOSPITAL ARRANGEMENTS.

The first effects of the war will be felt undoubtedly in East Anglia, and it is interesting to note what arrangements are being made in the various hospitals for dealing with the wounded.

Cambridge.

The chief hospital and depôt of the Voluntary Aid Detachments is at Cambridge. Arrangements on a vast scale will doubtless be made here. The medical staff is drawn from Addenbrooke's, assisted by members of the staffs of other East Anglian hospitals, including two from the East Suffolk Hospital at Ipswich.

No doubt the selection of Cambridge for the depôt for East Anglia was one, but the distance from the coast is considerable, and the difficulty of transit much more tedious than the journey to London.

Yarmouth.

A Red Cross Hospital has been established at Yarmouth, Sir Savile Crossley, Bart., having given £100 towards its equipment, and Lady Crossley and her friends a further £40. Mrs. C. S. Orde has promised a supply of articles for use in the hospital, and fully-equipped beds have been given by Messrs. Palmers, Arnolds and Bonings, of Yarmouth. Altogether fifty beds, with an ample supply of nurses and cooks, have been organised. The Red Cross Society has at present the use of the Sailors' Home on the Marine Parade, and of the Drill Hall, for hospital purposes.

Norfolk and Suffolk.

The managers of the Norfolk and Norwich Hospital have taken the lead by calling the public's attention to the probability that a considerable number of wounded may require accommodation and treatment in the Eastern Counties. Four hospital tents, with accommodation for sixty-four patients, have been given by an anonymous donor, and will be erected immediately in the grounds of the Norfolk and Norwich Hospital, Norwich. Donations are urgently needed for the equipment and maintenance of these tents, and for the further accommodation which will be necessary. The

British Red Cross Society, through Mrs. Astley, of Little Plumstead Hall, Norwich, has made an appeal to all men and women in the division who are willing to give their services and form Voluntary Aid Detachments. Mrs. Barclay, Commandant, Red Cross Society, Cromer Women's Detachment, has issued a manifesto asking for help, and also for recruits for a working party, meeting at the Fletcher Convalescent Home, for making night-shirts, bed-jackets, and other numerous garments which will be required. A Cromer Women's Detachment has been organising for four years, and Mrs. Barclay is not unnaturally anxious to be fully prepared to meet every requirement entailed by rendering efficient help to our soldiers and bluejackets. We believe and hope that the inhabitants of Cromer and the district will generously and liberally respond to Mrs. Barclay's appeal, by sending her handsome contributions in money as well as materials and articles useful in hospital. She also needs new recruits quickly.

Ipswich.

The East Suffolk Hospital at Ipswich, in the ordinary way, deals with cases coming in from a seaboard extending from Harwich Harbour on the south to a point between Aldeburgh and Southwold on the north. It is not surprising, therefore, that the board of management, recognising the importance of the strategical position which its district includes, took immediate steps to cope with any emergency. Arrangements have been made for placing the whole of the men's surgical ward, the isolation block (transformed into a surgical block), and other small wards at the disposal of the wounded soldiers and sailors—in all some 101 beds have been allocated for this purpose, and the fact communicated to the authorities, who have conveyed their thanks to the hospital board for its prompt action. The Ipswich Nurses' Home, under the capable management of Miss Newton, who is likely to be the matron of the chief East Anglian hospital at Cambridge, has also arranged thirty-eight beds for the wounded, while the Red Cross and other kindred societies have a number of beds and equipment ready—in all Ipswich has 500 beds available.

These arrangements, however, will probably not be called into requisition until the Royal Naval Hospital at Shotley, the headquarters of the Harwich Torpedo Destroyer Flotilla, is full.

Felixstowe.

At Felixstowe the troops have commandeered several public and private buildings—the Suffolk Convalescent Home, St. Mary's Children's Home, and the London Convalescent Home—for military hospital purposes, while the Pier Hotel, Felixstowe, has been converted into a hospital for the Red Cross Voluntary Aid Detachment.

The great difficulty with the Red Cross Voluntary Aid Detachments is still the lack of members with actual hospital training. The board of management of the East Suffolk Hospital during the last fortnight has been besieged with applications from ladies desirous of having a few weeks' practical casualty and theatre work before departing for their various centres.

